# Supplementary material for: Retrospective Cohort Study: Extracting Coexisting Background Breast-Lesion Features from Stage I–III Invasive Breast Cancer
Source: Cancers (Basel). 2026 Jun 17;18(12):1965. doi: 10.3390/cancers18121965 (PMC13297060; doi:10.3390/cancers18121965)

SUPPLEMENTARY MATERIAL

Supplementary Table S1. Comparison of breast features detected by core needle biopsy (green) and excision (orange) procedures. The table below shows the concordance between lesion presence on core needle biopsy (CNB) and subsequent surgical excision in 1,398 patients with both records. Cohen's kappa (κ) is reported to assess agreement beyond chance. κ values closer to 1 indicate stronger agreement.

Supplementary Table S2. Association between breast features and tumor characteristics in 3,164 breast cancer cases with reports from core needle biopsies. Odds ratios (ORs) and 95% confidence intervals (CIs) are presented from multinomial logistic regression models evaluating the association between breast features and tumor characteristics at diagnosis. Each OR reflects the odds of having a specific tumor characteristic versus a reference category, given the presence of a breast feature. All models are adjusted for potential confounders, including age at diagnosis, year of diagnosis, ethnicity, family history of breast cancer, menopausal status, and parity. Statistically significant associations ($p < 0.05$) are indicated in bold, those that remain significant after Benjamini-Hochberg correction are denoted by *. LCIS: Lobular carcinoma in situ; ALH: Atypical lobular hyperplasia; CCC: Columnar cell change; UDH: Usual ductal hyperplasia; DCIS: Ductal carcinoma in situ; ADH: Atypical ductal hyperplasia; FEA: Flat epithelial atypia

Supplementary Table S3. Association between breast features and tumor characteristics in 1,756 breast cancer cases with reports from excision procedures, diagnosed before 2010. Odds ratios (ORs) and 95% confidence intervals (CIs) are presented from multinomial logistic regression models evaluating the association between breast features and tumor characteristics at diagnosis. Each OR reflects the odds of having a specific tumor characteristic versus a reference category, given the presence of a feature. All models are adjusted for potential confounders, including age at diagnosis, year of diagnosis, ethnicity, family history of breast cancer, menopausal status, and parity. Statistically significant associations ($p < 0.05$) are indicated in bold, those that remain significant after Benjamini-Hochberg correction are denoted by *. LCIS: Lobular carcinoma in situ; ALH: Atypical lobular hyperplasia; CCC: Columnar cell change; UDH: Usual ductal hyperplasia; DCIS: Ductal carcinoma in situ; ADH: Atypical ductal hyperplasia; FEA: Flat epithelial atypia

Supplementary Table S4. Association between breast features and tumor characteristics in 2,232 breast cancer cases with reports from excision procedures, diagnosed 2010 and after. Odds ratios (ORs) and 95% confidence intervals (CIs) are presented from multinomial logistic regression models evaluating the association between breast features and tumor characteristics at diagnosis. Each OR reflects the odds of having a specific tumor characteristic versus a reference category, given the presence of a feature. All models are adjusted for potential confounders, including age at diagnosis, year of diagnosis, ethnicity, family history of breast cancer, menopausal status, and parity. Statistically significant associations ($p < 0.05$) are indicated in bold, those that remain significant after Benjamini-Hochberg correction are denoted by *. LCIS: Lobular carcinoma in situ; ALH: Atypical lobular hyperplasia; CCC: Columnar cell change; UDH: Usual ductal hyperplasia; DCIS: Ductal carcinoma in situ; ADH: Atypical ductal hyperplasia; FEA: Flat epithelial atypia

Supplementary Table S5. Association between coexisting features and 10-year overall survival in 3,164 breast cancer patients with CNB reports (613 events). Hazard ratios (HRs) with 95% confidence intervals (CIs) are presented from Cox proportional hazards models evaluating the impact of coexisting breast features on 10-year overall survival among breast cancer patients. Statistically significant associations ($p < 0.05$) are indicated in bold, those that remain significant after Benjamini-Hochberg correction are denoted by *. a) Adjusted for age at diagnosis and year of diagnosis. b) Model one further adjusted for menstruation status, ethnicity, family history of cancer and parity. c) Model two further adjusted for tumour characteristics: stage and subtype.

Supplementary Table S6. Association between coexisting features and 10-year overall survival in 1,756 breast cancer cases with reports from excision procedures, diagnosed before 2010. Hazard ratios (HRs) with 95% confidence intervals (CIs) are presented from Cox proportional hazards models evaluating the impact of coexisting breast features on 10-year overall survival among breast cancer patients. Statistically significant associations ($p < 0.05$) are indicated in bold, those that remain significant after Benjamini-Hochberg correction are denoted by *. a) Adjusted for age at diagnosis and year of diagnosis. b) Model one further adjusted for menstruation status, ethnicity, family history of cancer and parity. c) Model two further adjusted for tumour characteristics: stage and subtype.

Supplementary Table S7. Association between coexisting features and 10-year overall survival in 2,232 breast cancer cases with reports from excision procedures, diagnosed 2010 and after. Hazard ratios (HRs) with 95% confidence intervals (CIs) are presented from Cox proportional hazards models evaluating the impact of coexisting breast features on 10-year overall survival among breast cancer patients. Statistically significant associations ($p < 0.05$) are indicated in bold, those that remain significant after Benjamini-Hochberg correction are denoted by *. a) Adjusted for age at diagnosis and year of diagnosis. b) Model one further adjusted for menstruation status, ethnicity, family history of cancer and parity. c) Model two further adjusted for tumour characteristics: stage and subtype.

Supplementary Table S8. Association between coexisting features and 10-year overall survival in 1,604 breast cancer cases with reports from excision procedures diagnosed with Stage I breast cancer. Hazard ratios (HRs) with 95% confidence intervals (CIs) are presented from Cox proportional hazards models evaluating the impact of coexisting

breast features on 10-year overall survival among breast cancer patients. Statistically significant associations ($p < 0.05$) are indicated in bold, those that remain significant after Benjamini-Hochberg correction are denoted by *. a) Adjusted for age at diagnosis and year of diagnosis. b) Model one further adjusted for menstruation status, ethnicity, family history of cancer and parity. c) Model two further adjusted for tumour characteristics: subtype.

Supplementary Table S9. Association between coexisting features and 10-year overall survival in 1,634 breast cancer cases with reports from excision procedures diagnosed with Stage II breast cancer. Hazard ratios (HRs) with 95% confidence intervals (CIs) are presented from Cox proportional hazards models evaluating the impact of coexisting breast features on 10-year overall survival among breast cancer patients. Statistically significant associations ($p < 0.05$) are indicated in bold, those that remain significant after Benjamini-Hochberg correction are denoted by *. a) Adjusted for age at diagnosis and year of diagnosis. b) Model one further adjusted for menstruation status, ethnicity, family history of cancer and parity. c) Model two further adjusted for tumour characteristics: subtype.

Supplementary Table S10. Association between coexisting features and 10-year overall survival in 750 breast cancer cases with reports from excision procedures diagnosed with Stage III breast cancer. Hazard ratios (HRs) with 95% confidence intervals (CIs) are presented from Cox proportional hazards models evaluating the impact of coexisting breast features on 10-year overall survival among breast cancer patients. Statistically significant associations ($p < 0.05$) are indicated in bold, those that remain significant after Benjamini-Hochberg correction are denoted by *. a) Adjusted for age at diagnosis and year of diagnosis. b) Model one further adjusted for menstruation status, ethnicity, family history of cancer and parity. c) Model two further adjusted for tumour characteristics: subtype.

Supplementary Table S11. Association between tumor stage and presence/number of breast features, using 3,988 breast cancer cases with reports from excision procedures.

Supplementary Figure S1. Flowchart of how analytical datasets were derived.

Supplementary Figure S2. Distribution of procedure date relative to diagnosis date.

Supplementary Figure S3. Cluster membership of breast features based on Pearson correlation hierarchical clustering for 3,988 records from excisions. **B)** Sensitivity analysis: Jaccard distance with complete linkage (Adjusted Rand Index vs. Panel A = 0.574). The lobular track (LCIS, ALH) is stable across both methods. Pre-malignant and high-risk ductal lesions (DCIS, intraductal papilloma) shift from the high-risk ductal cluster (A) to the benign cluster (B), reflecting Jaccard's exclusion of joint absences which masks the epidemiological rarity these lesions share across benign screening records.

Supplementary Table S1. Comparison of breast features detected by core needle biopsy (green) and excision (orange) procedures. The table below shows the concordance between lesion presence on core needle biopsy (CNB) and subsequent surgical excision in 1,398 patients with both records. Cohen's kappa (κ) is reported to assess agreement beyond chance. κ values closer to 1 indicate stronger agreement.

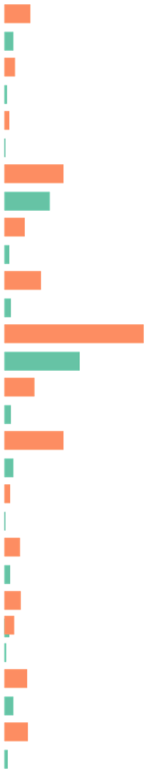

	Present (n, %)	CNB (+) & Excision (+)	CNB (+) & Excision (−)	CNB (−) & Excision (+)	CNB (−) & Excision (−)	Cohen's κ
Apocrine metaplasia		22 (1.6%)	35 (2.5%)	142 (10.2%)	1,199 (85.8%)	0.148
Atypical ductal hyperplasia (ADH)		2 (0.1%)	14 (1.0%)	65 (4.6%)	1,317 (94.2%)	0.03
Atypical lobular hyperplasia (ALH)		1 (0.1%)	8 (0.6%)	30 (2.1%)	1,359 (97.2%)	0.04
Calcification		134 (9.6%)	157 (11.2%)	243 (17.4%)	864 (61.8%)	0.217
Columnar cell change		3 (0.2%)	32 (2.3%)	127 (9.1%)	1,236 (88.4%)	-0.003
Cyst		16 (1.1%)	29 (2.1%)	216 (15.5%)	1,137 (81.3%)	0.065
DCIS		386 (27.6%)	95 (6.8%)	497 (35.6%)	420 (30.0%)	0.217
Fibroadenoma		18 (1.3%)	24 (1.7%)	175 (12.5%)	1,181 (84.5%)	0.109
Fibrocystic change		21 (1.5%)	37 (2.6%)	355 (25.4%)	985 (70.5%)	0.027
Flat epithelial atypia (FEA)		1 (0.1%)	7 (0.5%)	39 (2.8%)	1,351 (96.6%)	0.032
Intraductal papilloma		8 (0.6%)	29 (2.1%)	91 (6.5%)	1,270 (90.8%)	0.082
LCIS		24 (1.7%)	7 (0.5%)	79 (5.7%)	1,288 (92.1%)	0.336
Radial scar or complex sclerosing lesion		6 (0.4%)	7 (0.5%)	58 (4.1%)	1,327 (94.9%)	0.143
Sclerosing adenosis		9 (0.6%)	51 (3.6%)	135 (9.7%)	1,203 (86.1%)	0.029
Usual ductal hyperplasia		5 (0.4%)	19 (1.4%)	146 (10.4%)	1,228 (87.8%)	0.028

Supplementary Table S2. Association between breast features and tumor characteristics in 3,164 breast cancer cases with reports from core needle biopsies. Odds ratios (ORs) and 95% confidence intervals (CIs) are presented from multinomial logistic regression models evaluating the association between breast features and tumor characteristics at diagnosis. Each OR reflects the odds of having a specific tumor characteristic versus a reference category, given the presence of a breast feature. All models are adjusted for potential confounders, including age at diagnosis, year of diagnosis, ethnicity, family history of breast cancer, menopausal status, and parity. Statistically significant associations (p < 0.05) are indicated in bold, those that remain significant after Benjamini-Hochberg correction are denoted by *. LCIS: Lobular carcinoma in situ; ALH: Atypical lobular hyperplasia; CCC: Columnar cell change; UDH: Usual ductal hyperplasia; DCIS: Ductal carcinoma in situ; ADH: Atypical ductal hyperplasia; FEA: Flat epithelial atypia

Reference	Stage I (n=801)		ER-pos (n=2,357)	PR-pos (n=2,005)	Tumor size <2cm (n=1,115)		Well-differentiated (n=340)		Nodal status neg (n=1,441)	Luminal A (n=1,824)		
	Stage II (n=1,537)	Stage III (n=826)	ER-neg (n=746)	PR-neg (n=1,096)	2-5cm (n=1,395)	>5cm (n=259)	Moderately- (n=1,174)	Poorly- (n=1,376)	Positive (n=1,125)	Luminal B (n=559)	HER2-enriched (n=291)	Triple-neg (n=335)
Lobular neoplasia												
LCIS	**0.51 (0.28, 0.91)***	0.58 (0.29, 1.15)	**0.30 (0.12, 0.76)***	0.71 (0.40, 1.28)	0.96 (0.52, 1.77)	1.59 (0.66, 3.83)	1.03 (0.48, 2.18)	**0.33 (0.14, 0.78)***	1.07 (0.59, 1.93)	0.47 (0.21, 1.06)	**0.13 (0.02, 0.97)**	**0.22 (0.05, 0.92)**
ALH	0.36 (0.10, 1.31)	0.17 (0.02, 1.44)	0.31 (0.04, 2.47)	0.19 (0.02, 1.47)	0.43 (0.11, 1.74)	1.56 (0.31, 7.90)	0.20 (0.03, 1.19)	0.32 (0.07, 1.45)	0.53 (0.14, 2.11)	0.39 (0.05, 3.13)	-	0.61 (0.08, 4.94)
Any (binary)	**0.49 (0.28, 0.86)***	0.55 (0.28, 1.06)	**0.33 (0.14, 0.77)***	0.67 (0.38, 1.18)	0.87 (0.49, 1.56)	1.81 (0.82, 4.00)	0.87 (0.43, 1.74)	**0.33 (0.15, 0.73)***	1.09 (0.63, 1.90)	**0.43 (0.19, 0.96)**	**0.12 (0.02, 0.87)**	**0.31 (0.09, 0.99)**
Any (continuous)	**0.52 (0.32, 0.86)***	**0.54 (0.29, 0.99)**	**0.34 (0.15, 0.77)***	0.64 (0.38, 1.09)	0.86 (0.51, 1.43)	1.46 (0.72, 2.97)	0.85 (0.46, 1.57)	**0.37 (0.18, 0.75)***	0.96 (0.58, 1.59)	0.50 (0.24, 1.02)	**0.13 (0.02, 0.92)**	**0.32 (0.10, 0.99)**
Benign or non-atypical proliferative breast changes												
Fibroadenoma	1.3 (0.84, 2.10)	0.57 (0.30, 1.09)	0.98 (0.62, 1.56)	0.86 (0.56, 1.30)	1.14 (0.76, 1.73)	0.32 (0.10, 1.03)	0.93 (0.50, 1.73)	0.73 (0.39, 1.36)	0.93 (0.61, 1.43)	1.12 (0.68, 1.84)	1.29 (0.69, 2.38)	0.68 (0.32, 1.43)
Calcification	**0.68 (0.55, 0.84)***	**0.71 (0.56, 0.91)***	**0.65 (0.51, 0.81)***	**0.77 (0.63, 0.93)***	0.83 (0.68, 1.01)	**0.61 (0.41, 0.89)***	1.01 (0.76, 1.35)	**0.63 (0.47, 0.85)***	1.17 (0.96, 1.43)	1.23 (0.98, 1.55)	1.10 (0.81, 1.50)	**0.40 (0.27, 0.59)***
Cyst	**0.5 (0.33, 0.85)***	**0.36 (0.18, 0.69)***	0.73 (0.42, 1.28)	0.69 (0.42, 1.12)	0.77 (0.47, 1.25)	0.85 (0.37, 1.96)	0.70 (0.38, 1.27)	**0.33 (0.17, 0.64)***	**0.42 (0.24, 0.73)***	0.66 (0.35, 1.24)	0.50 (0.20, 1.26)	0.74 (0.35, 1.58)
Apocrine metaplasia	**1.61 (1.01, 2.56)**	0.94 (0.52, 1.71)	**4.35 (2.98, 6.36)***	**2.10 (1.45, 3.06)***	1.03 (0.69, 1.54)	0.74 (0.33, 1.66)	1.74 (0.77, 3.93)	**2.35 (1.06, 5.20)***	0.77 (0.50, 1.18)	**2.61 (1.57, 4.34)***	**4.87 (2.85, 8.33)***	**4.86 (2.90, 8.16)***
CCC	**0.55 (0.31, 0.96)**	**0.50 (0.25, 1.00)**	**0.32 (0.13, 0.74)***	**0.31 (0.15, 0.63)***	0.72 (0.41, 1.27)	1.14 (0.49, 2.68)	**0.44 (0.23, 0.85)**	**0.25 (0.12, 0.51)***	0.63 (0.35, 1.13)	0.82 (0.43, 1.57)	**0.13 (0.02, 0.95)**	**0.22 (0.05, 0.92)**
UDH	1.29 (0.69, 2.42)	0.90 (0.42, 1.96)	0.89 (0.48, 1.63)	**0.49 (0.27, 0.91)**	0.90 (0.51, 1.59)	1.10 (0.44, 2.76)	0.48 (0.22, 1.02)	0.59 (0.29, 1.21)	0.94 (0.53, 1.69)	0.96 (0.49, 1.86)	1.41 (0.67, 2.98)	0.54 (0.19, 1.54)
Sclerosing adenosis	0.75 (0.51, 1.10)	**0.47 (0.28, 0.79)***	0.93 (0.61, 1.41)	0.79 (0.54, 1.15)	1.14 (0.78, 1.66)	0.66 (0.29, 1.48)	0.61 (0.37, 1.01)	**0.50 (0.30, 0.84)***	**0.59 (0.39, 0.88)**	0.79 (0.48, 1.28)	0.94 (0.51, 1.71)	0.62 (0.32, 1.21)
Fibrocystic change	0.82 (0.54, 1.25)	0.68 (0.41, 1.14)	0.98 (0.64, 1.49)	0.97 (0.66, 1.42)	0.69 (0.46, 1.04)	0.85 (0.44, 1.68)	0.61 (0.36, 1.05)	**0.53 (0.31, 0.91)***	0.74 (0.49, 1.12)	0.93 (0.57, 1.52)	1.10 (0.60, 2.02)	0.96 (0.53, 1.74)
Any (binary)	**0.71 (0.59, 0.85)***	**0.65 (0.52, 0.81)***	0.89 (0.74, 1.08)	**0.83 (0.71, 0.98)**	**0.84 (0.70, 0.99)**	**0.67 (0.49, 0.93)**	0.92 (0.72, 1.19)	**0.64 (0.49, 0.82)***	0.97 (0.81, 1.15)	1.16 (0.94, 1.42)	**1.33 (1.02, 1.73)**	**0.66 (0.50, 0.87)***
Any (continuous)	**0.8 (0.80, 0.98)***	**0.77 (0.68, 0.88)***	0.95 (0.86, 1.06)	**0.89 (0.81, 0.99)**	0.93 (0.84, 1.03)	**0.81 (0.66, 0.98)**	0.89 (0.78, 1.02)	**0.75 (0.65, 0.86)***	0.92 (0.83, 1.02)	1.06 (0.95, 1.19)	1.09 (0.95, 1.26)	**0.81 (0.68, 0.96)***
Early neoplastic, papillary and complex sclerosing lesions												
DCIS	**0.67 (0.56, 0.80)***	**0.52 (0.42, 0.65)***	**0.72 (0.59, 0.87)***	**0.80 (0.67, 0.94)***	**0.73 (0.61, 0.86)***	**0.53 (0.38, 0.73)***	0.83 (0.65, 1.08)	**0.60 (0.46, 0.78)***	1.00 (0.84, 1.19)	1.16 (0.94, 1.42)	1.07 (0.82, 1.40)	**0.43 (0.31, 0.59)***
ADH	0.6 (0.25, 1.52)	0.25 (0.05, 1.16)	0.52 (0.15, 1.77)	0.61 (0.22, 1.67)	0.77 (0.29, 2.01)	1.64 (0.44, 6.17)	**0.35 (0.12, 0.97)**	**0.18 (0.06, 0.57)***	0.36 (0.12, 1.11)	0.40 (0.09, 1.77)	0.39 (0.05, 2.96)	0.69 (0.16, 3.03)
FEA	**0.24 (0.06, 0.96)**	-	0.87 (0.18, 4.24)	0.26 (0.03, 2.07)	**0.12 (0.01, 0.95)**	-	0.49 (0.11, 2.07)	**0.08 (0.01, 0.73)***	-	1.50 (0.37, 6.08)	-	-
Radial scar/complex sclerosing	0.66 (0.24, 1.80)	0.44 (0.11, 1.76)	-	0.39 (0.11, 1.35)	1.27 (0.49, 3.33)	0.60 (0.07, 5.02)	1.00 (0.28, 3.64)	0.33 (0.07, 1.48)	0.98 (0.36, 2.62)	0.18 (0.02, 1.33)	**-**	-
Intraductal papilloma	**0.51 (0.31, 0.86)***	**0.28 (0.13, 0.61)***	1.29 (0.75, 2.21)	1.30 (0.79, 2.15)	0.59 (0.34, 1.02)	0.43 (0.13, 1.43)	**0.36 (0.19, 0.69)***	**0.35 (0.18, 0.66)***	**0.52 (0.29, 0.93)**	0.45 (0.19, 1.07)	0.89 (0.37, 2.13)	1.48 (0.75, 2.93)
Any (binary)	**0.64 (0.53, 0.76)***	**0.49 (0.40, 0.61)***	**0.73 (0.61, 0.88)***	**0.81 (0.69, 0.96)***	**0.73 (0.62, 0.86)***	**0.51 (0.37, 0.70)***	0.80 (0.62, 1.04)	**0.57 (0.45, 0.74)***	0.95 (0.80, 1.13)	1.08 (0.88, 1.33)	1.04 (0.80, 1.36)	**0.45 (0.34, 0.61)***
Any (continuous)	**0.66 (0.56, 0.78)***	**0.50 (0.41, 0.61)***	**0.75 (0.63, 0.89)***	**0.82 (0.71, 0.95)***	**0.73 (0.63, 0.85)***	**0.56 (0.41, 0.75)***	**0.75 (0.60, 0.93)**	**0.54 (0.43, 0.67)***	0.92 (0.78, 1.07)	1.03 (0.86, 1.23)	0.98 (0.77, 1.24)	**0.50 (0.38, 0.66)***

Supplementary Table S3. Association between breast features and tumor characteristics in 1,756 breast cancer cases with reports from excision procedures, diagnosed before 2010. Odds ratios (ORs) and 95% confidence intervals (CIs) are presented from multinomial logistic regression models evaluating the association between breast features and tumor characteristics at diagnosis. Each OR reflects the odds of having a specific tumor characteristic versus a reference category, given the presence of a feature. All models are adjusted for potential confounders, including age at diagnosis, year of diagnosis, ethnicity, family history of breast cancer, menopausal status, and parity. Statistically significant associations ($p < 0.05$) are indicated in bold, those that remain significant after Benjamini-Hochberg correction are denoted by *. LCIS: Lobular carcinoma in situ; ALH: Atypical lobular hyperplasia; CCC: Columnar cell change; UDH: Usual ductal hyperplasia; DCIS: Ductal carcinoma in situ; ADH: Atypical ductal hyperplasia; FEA: Flat epithelial atypia

Reference	Stage I (n=621)		ER-pos (n=1,098)	PR-pos (n=950)	Tumor size <2cm (n=754)		Well-differentiated (n=250)		Nodal status neg (n=881)	Luminal A (n=787)		
	Stage II (n=798)	Stage III (n=337)	ER-neg (n=520)	PR-neg (n=657)	2-5cm (n=638)	>5cm (n=124)	Moderately- (n=636)	Poorly- (n=764)	Positive (n=609)	Luminal B (n=223)	HER2-enriched (n=171)	Triple-neg (n=193)
Lobular neoplasia												
LCIS	0.69 (0.42, 1.12)	0.73 (0.39, 1.37)	**0.40 (0.22, 0.73)***	**0.50 (0.30, 0.82)***	0.94 (0.57, 1.55)	**2.18 (1.07, 4.45)**	1.13 (0.63, 2.04)	**0.26 (0.12, 0.54)***	0.82 (0.50, 1.35)	**0.27 (0.11, 0.68)**	**0.20 (0.06, 0.66)***	**0.30 (0.12, 0.77)***
ALH	0.46 (0.21, 1.01)	0.34 (0.10, 1.17)	**0.23 (0.07, 0.76)**	0.49 (0.22, 1.12)	**0.40 (0.16, 0.96)**	0.67 (0.15, 3.00)	**0.31 (0.12, 0.77)***	**0.20 (0.07, 0.53)***	0.88 (0.40, 1.94)	1.23 (0.48, 3.18)	0.27 (0.04, 2.07)	0.20 (0.03, 1.55)
Any (binary)	**0.61 (0.39, 0.95)**	0.56 (0.30, 1.03)	**0.33 (0.18, 0.58)***	**0.49 (0.31, 0.78)***	0.77 (0.48, 1.22)	1.61 (0.81, 3.21)	0.78 (0.46, 1.30)	**0.20 (0.11, 0.39)***	0.76 (0.48, 1.21)	**0.37 (0.17, 0.78)**	**0.18 (0.05, 0.57)***	**0.26 (0.10, 0.65)***
Any (continuous)	**0.67 (0.46, 0.98)**	0.66 (0.40, 1.10)	**0.42 (0.25, 0.69)***	**0.56 (0.38, 0.83)***	0.78 (0.53, 1.16)	1.47 (0.84, 2.58)	0.83 (0.54, 1.27)	**0.29 (0.17, 0.51)***	0.86 (0.59, 1.27)	**0.54 (0.30, 0.99)**	**0.28 (0.10, 0.73)***	**0.35 (0.16, 0.77)***
Benign or non-atypical proliferative breast changes												
Fibroadenoma	0.73 (0.50, 1.06)	0.79 (0.49, 1.27)	0.71 (0.48, 1.05)	0.78 (0.54, 1.12)	0.91 (0.63, 1.30)	**0.21 (0.07, 0.69)***	**0.57 (0.35, 0.92)**	**0.62 (0.39, 0.99)**	**0.61 (0.41, 0.90)***	0.83 (0.50, 1.40)	0.52 (0.27, 1.03)	0.85 (0.50, 1.46)
Calcification	**0.56 (0.43, 0.73)***	**0.32 (0.21, 0.48)***	0.88 (0.67, 1.16)	0.92 (0.71, 1.19)	**0.55 (0.41, 0.72)***	**0.49 (0.28, 0.85)***	**0.61 (0.42, 0.87)***	**0.61 (0.43, 0.86)***	**0.67 (0.50, 0.88)***	0.87 (0.59, 1.28)	1.09 (0.72, 1.63)	0.70 (0.45, 1.08)
Cyst	0.80 (0.57, 1.11)	0.63 (0.40, 1.00)	1.02 (0.73, 1.43)	0.91 (0.66, 1.26)	0.88 (0.63, 1.23)	0.71 (0.37, 1.36)	0.70 (0.45, 1.09)	0.73 (0.47, 1.12)	**0.63 (0.45, 0.90)***	0.66 (0.40, 1.11)	0.82 (0.48, 1.40)	0.96 (0.58, 1.56)
Apocrine metaplasia	**0.64 (0.43, 0.95)***	**0.49 (0.28, 0.86)***	1.18 (0.80, 1.74)	0.79 (0.53, 1.16)	**0.64 (0.42, 0.96)**	0.72 (0.34, 1.52)	0.73 (0.44, 1.22)	0.65 (0.39, 1.08)	0.91 (0.61, 1.36)	0.80 (0.44, 1.43)	0.88 (0.47, 1.65)	0.93 (0.52, 1.69)
CCC	**0.53 (0.33, 0.84)***	**0.50 (0.26, 0.95)**	0.80 (0.50, 1.30)	0.78 (0.50, 1.23)	0.81 (0.51, 1.27)	0.47 (0.16, 1.34)	**0.48 (0.27, 0.85)***	**0.42 (0.24, 0.74)***	**0.56 (0.34, 0.92)**	0.78 (0.41, 1.50)	0.70 (0.33, 1.52)	0.90 (0.46, 1.77)
UDH	**0.62 (0.42, 0.92)***	**0.16 (0.07, 0.37)***	0.96 (0.63, 1.45)	0.71 (0.47, 1.07)	**0.60 (0.39, 0.91)**	**0.30 (0.11, 0.86)**	**0.53 (0.31, 0.88)***	**0.51 (0.31, 0.85)***	**0.52 (0.33, 0.81)***	0.78 (0.43, 1.40)	0.94 (0.50, 1.76)	0.67 (0.34, 1.30)
Sclerosing adenosis	**0.53 (0.36, 0.79)***	**0.39 (0.21, 0.70)***	1.25 (0.85, 1.85)	0.98 (0.66, 1.44)	**0.51 (0.33, 0.78)***	**0.40 (0.17, 0.97)**	**0.46 (0.28, 0.77)***	**0.49 (0.30, 0.80)***	0.71 (0.46, 1.08)	0.83 (0.46, 1.50)	1.42 (0.81, 2.49)	1.08 (0.59, 1.96)
Fibrocystic change	0.79 (0.62, 1.00)	**0.49 (0.34, 0.69)***	0.86 (0.67, 1.11)	0.82 (0.64, 1.04)	0.81 (0.63, 1.04)	**0.47 (0.28, 0.81)***	**0.65 (0.46, 0.90)***	**0.60 (0.44, 0.83)***	**0.65 (0.50, 0.84)***	0.87 (0.61, 1.24)	0.78 (0.52, 1.18)	0.81 (0.55, 1.18)
Any (binary)	**0.70 (0.56, 0.87)***	**0.46 (0.35, 0.61)***	0.92 (0.74, 1.14)	0.85 (0.70, 1.05)	**0.74 (0.59, 0.91)***	**0.45 (0.30, 0.68)***	**0.71 (0.53, 0.96)**	**0.68 (0.51, 0.92)***	**0.67 (0.54, 0.83)***	0.96 (0.71, 1.30)	0.92 (0.66, 1.29)	0.88 (0.63, 1.21)
Any (continuous)	**0.86 (0.80, 0.92)***	**0.73 (0.65, 0.82)***	0.97 (0.90, 1.05)	0.94 (0.87, 1.01)	**0.88 (0.81, 0.95)***	**0.74 (0.62, 0.88)***	**0.83 (0.76, 0.91)***	**0.82 (0.75, 0.90)***	**0.85 (0.78, 0.92)***	0.93 (0.83, 1.03)	0.95 (0.85, 1.07)	0.93 (0.83, 1.05)
Early neoplastic, papillary and complex sclerosing lesions												
DCIS	**0.53 (0.42, 0.66)***	**0.58 (0.43, 0.77)***	0.89 (0.72, 1.11)	**0.75 (0.61, 0.93)***	**0.64 (0.51, 0.80)***	**0.52 (0.35, 0.77)***	1.10 (0.81, 1.50)	0.89 (0.66, 1.20)	1.13 (0.90, 1.41)	1.11 (0.80, 1.53)	**1.97 (1.32, 2.95)***	**0.51 (0.37, 0.71)***
ADH	**0.53 (0.31, 0.93)***	**0.21 (0.07, 0.61)***	**0.39 (0.19, 0.81)**	0.68 (0.38, 1.20)	**0.53 (0.30, 0.94)**	0.15 (0.02, 1.09)	0.56 (0.30, 1.02)	**0.19 (0.09, 0.40)***	**0.52 (0.28, 0.97)**	0.82 (0.39, 1.74)	0.50 (0.18, 1.43)	0.32 (0.10, 1.05)
FEA	1.15 (0.25, 5.32)	0.68 (0.07, 6.84)	1.30 (0.30, 5.57)	0.90 (0.21, 3.88)	0.75 (0.17, 3.25)	-	0.45 (0.06, 3.27)	0.54 (0.09, 3.32)	0.53 (0.10, 2.74)	0.87 (0.10, 7.88)	-	3.26 (0.69, 15.35)
Radial scar/complex sclerosing	0.61 (0.31, 1.19)	0.51 (0.20, 1.31)	0.57 (0.27, 1.21)	0.51 (0.25, 1.03)	0.98 (0.51, 1.87)	0.84 (0.24, 2.94)	**0.37 (0.17, 0.81)***	**0.39 (0.18, 0.84)***	**0.45 (0.21, 0.94)**	0.70 (0.26, 1.87)	0.54 (0.16, 1.81)	0.68 (0.23, 2.00)
Intraductal papilloma	0.68 (0.37, 1.24)	0.75 (0.36, 1.60)	0.68 (0.36, 1.29)	0.60 (0.33, 1.10)	0.88 (0.50, 1.58)	1.00 (0.37, 2.76)	0.97 (0.44, 2.11)	0.78 (0.36, 1.70)	0.95 (0.52, 1.73)	0.89 (0.40, 1.97)	0.86 (0.35, 2.11)	0.34 (0.10, 1.14)
Any (binary)	**0.51 (0.41, 0.64)***	**0.57 (0.43, 0.76)***	0.89 (0.71, 1.11)	**0.74 (0.60, 0.91)***	**0.63 (0.50, 0.79)***	**0.53 (0.35, 0.79)***	1.08 (0.79, 1.47)	0.84 (0.62, 1.13)	1.11 (0.89, 1.39)	1.02 (0.74, 1.42)	**1.81 (1.20, 2.72)***	**0.50 (0.36, 0.70)***
Any (continuous)	**0.60 (0.50, 0.71)***	**0.60 (0.48, 0.75)***	**0.82 (0.69, 0.97)**	**0.75 (0.63, 0.88)***	**0.71 (0.59, 0.85)***	**0.57 (0.41, 0.79)***	0.89 (0.70, 1.12)	**0.71 (0.56, 0.90)***	0.95 (0.80, 1.13)	1.00 (0.79, 1.27)	1.23 (0.95, 1.60)	**0.54 (0.40, 0.71)***

Supplementary Table S4. Association between breast features and tumor characteristics in 2,232 breast cancer cases with reports from excision procedures, diagnosed 2010 and after. Odds ratios (ORs) and 95% confidence intervals (CIs) are presented from multinomial logistic regression models evaluating the association between breast features and tumor characteristics at diagnosis. Each OR reflects the odds of having a specific tumor characteristic versus a reference category, given the presence of a feature. All models are adjusted for potential confounders, including age at diagnosis, year of diagnosis, ethnicity, family history of breast cancer, menopausal status, and parity. Statistically significant associations ($p < 0.05$) are indicated in bold, those that remain significant after Benjamini-Hochberg correction are denoted by *. LCIS: Lobular carcinoma in situ; ALH: Atypical lobular hyperplasia; CCC: Columnar cell change; UDH: Usual ductal hyperplasia; DCIS: Ductal carcinoma in situ; ADH: Atypical ductal hyperplasia; FEA: Flat epithelial atypia

Reference	Stage I (n=983)		ER-pos (n=1,773)	PR-pos (n=1,479)	Tumor size <2cm (n=1,090)		Well-differentiated (n=317)		Nodal status neg (n=1,373)	Luminal A (n=1,436)		
	Stage II (n=836)	Stage III (n=413)	ER-neg (n=444)	PR-neg (n=733)	2-5cm (n=872)	>5cm (n=160)	Moderately- (n=927)	Poorly- (n=910)	Positive (n=662)	Luminal B (n=334)	HER2-enriched (n=199)	Triple-neg (n=197)
Lobular neoplasia												
LCIS	0.92 (0.64, 1.32)	0.82 (0.51, 1.31)	**0.46 (0.27, 0.77)***	0.71 (0.49, 1.04)	0.89 (0.63, 1.28)	1.50 (0.86, 2.64)	1.50 (0.93, 2.43)	**0.50 (0.29, 0.86)***	0.90 (0.62, 1.31)	**0.47 (0.27, 0.81)***	0.50 (0.25, 1.01)	**0.45 (0.21, 0.94)**
ALH	**0.29 (0.14, 0.61)***	**0.27 (0.10, 0.70)***	**0.17 (0.04, 0.70)***	0.62 (0.31, 1.23)	**0.35 (0.18, 0.71)***	0.31 (0.07, 1.33)	0.80 (0.38, 1.69)	**0.34 (0.14, 0.82)***	**0.45 (0.21, 0.96)**	**0.28 (0.09, 0.92)**	0.17 (0.02, 1.23)	0.17 (0.02, 1.24)
Any (binary)	0.85 (0.60, 1.19)	0.76 (0.49, 1.18)	**0.41 (0.25, 0.68)***	**0.66 (0.46, 0.95)**	0.86 (0.62, 1.20)	1.29 (0.75, 2.22)	1.16 (0.76, 1.79)	**0.45 (0.28, 0.73)***	0.87 (0.61, 1.24)	**0.45 (0.27, 0.76)***	**0.42 (0.21, 0.85)**	**0.43 (0.21, 0.86)**
Any (continuous)	**0.75 (0.56, 1.00)**	**0.67 (0.46, 0.99)**	**0.44 (0.28, 0.70)***	**0.73 (0.54, 0.99)**	0.76 (0.57, 1.01)	1.06 (0.67, 1.70)	1.22 (0.84, 1.76)	**0.50 (0.32, 0.77)***	0.80 (0.59, 1.09)	**0.48 (0.30, 0.76)***	**0.47 (0.26, 0.88)**	**0.43 (0.23, 0.83)***
Benign or non-atypical proliferative breast changes												
Fibroadenoma	1.21 (0.92, 1.58)	**1.43 (1.04, 1.98)***	0.96 (0.72, 1.30)	0.90 (0.69, 1.16)	**1.32 (1.02, 1.70)**	1.47 (0.94, 2.28)	1.09 (0.75, 1.58)	1.18 (0.81, 1.71)	1.14 (0.88, 1.49)	0.74 (0.52, 1.05)	1.03 (0.69, 1.55)	0.72 (0.45, 1.14)
Calcification	**0.64 (0.52, 0.79)***	**0.77 (0.59, 1.00)**	0.94 (0.75, 1.19)	1.03 (0.85, 1.26)	**0.71 (0.58, 0.87)***	0.76 (0.52, 1.11)	1.27 (0.96, 1.69)	1.02 (0.76, 1.36)	**0.68 (0.54, 0.84)***	1.16 (0.90, 1.50)	1.28 (0.94, 1.76)	**0.65 (0.45, 0.93)**
Cyst	1.11 (0.87, 1.42)	1.00 (0.74, 1.35)	0.95 (0.73, 1.25)	0.82 (0.64, 1.04)	0.95 (0.75, 1.20)	1.44 (0.97, 2.14)	0.89 (0.64, 1.23)	0.81 (0.58, 1.12)	1.05 (0.83, 1.34)	0.92 (0.68, 1.26)	0.94 (0.64, 1.38)	0.93 (0.63, 1.38)
Apocrine metaplasia	0.76 (0.58, 1.00)	**0.46 (0.30, 0.69)***	**1.70 (1.28, 2.26)***	**1.36 (1.05, 1.77)**	**0.68 (0.52, 0.90)***	0.72 (0.43, 1.23)	1.15 (0.77, 1.74)	1.33 (0.89, 2.00)	**0.70 (0.52, 0.95)**	1.03 (0.71, 1.49)	1.47 (0.97, 2.23)	**1.72 (1.15, 2.56)***
CCC	**0.71 (0.53, 0.95)***	**0.50 (0.33, 0.76)***	0.95 (0.67, 1.33)	0.89 (0.67, 1.20)	**0.69 (0.52, 0.93)***	**0.55 (0.30, 1.00)**	**0.67 (0.47, 0.97)**	**0.51 (0.35, 0.75)***	**0.65 (0.47, 0.90)***	1.02 (0.70, 1.48)	0.99 (0.61, 1.60)	0.87 (0.53, 1.45)
UDH	0.82 (0.62, 1.07)	**0.50 (0.34, 0.75)***	0.81 (0.58, 1.13)	0.95 (0.72, 1.24)	0.85 (0.65, 1.11)	**0.43 (0.23, 0.81)***	0.89 (0.61, 1.29)	0.90 (0.62, 1.32)	**0.62 (0.46, 0.84)***	1.16 (0.82, 1.63)	0.87 (0.55, 1.38)	0.76 (0.47, 1.24)
Sclerosing adenosis	0.80 (0.59, 1.07)	**0.59 (0.39, 0.89)***	0.85 (0.60, 1.20)	0.92 (0.69, 1.23)	0.76 (0.56, 1.02)	0.73 (0.41, 1.29)	1.13 (0.74, 1.72)	1.11 (0.72, 1.69)	0.73 (0.52, 1.01)	1.28 (0.89, 1.82)	1.07 (0.67, 1.71)	0.66 (0.38, 1.16)
Fibrocystic change	**0.72 (0.59, 0.89)***	**0.69 (0.53, 0.91)***	0.95 (0.75, 1.20)	0.88 (0.72, 1.08)	**0.73 (0.60, 0.90)***	0.80 (0.54, 1.18)	0.87 (0.65, 1.15)	0.87 (0.66, 1.16)	**0.76 (0.61, 0.95)**	0.83 (0.63, 1.09)	1.01 (0.73, 1.41)	0.76 (0.53, 1.08)
Any (binary)	**0.82 (0.68, 0.99)**	0.84 (0.66, 1.08)	1.13 (0.92, 1.41)	1.12 (0.93, 1.34)	**0.78 (0.65, 0.94)***	0.94 (0.66, 1.33)	0.94 (0.72, 1.22)	0.93 (0.72, 1.22)	**0.79 (0.65, 0.96)**	1.02 (0.80, 1.31)	**1.46 (1.06, 2.00)**	0.91 (0.67, 1.23)
Any (continuous)	**0.92 (0.87, 0.97)***	**0.88 (0.82, 0.95)***	1.00 (0.94, 1.06)	0.98 (0.93, 1.04)	**0.92 (0.87, 0.97)***	0.93 (0.84, 1.03)	0.99 (0.92, 1.07)	0.97 (0.90, 1.05)	**0.91 (0.85, 0.96)***	1.00 (0.93, 1.07)	1.03 (0.95, 1.13)	0.93 (0.84, 1.02)
Early neoplastic, papillary and complex sclerosing lesions												
DCIS	**0.71 (0.59, 0.87)***	**0.74 (0.57, 0.94)***	0.92 (0.74, 1.15)	0.90 (0.74, 1.08)	**0.81 (0.67, 0.98)**	**0.51 (0.36, 0.72)***	1.19 (0.91, 1.55)	1.09 (0.83, 1.42)	0.98 (0.80, 1.20)	**1.43 (1.10, 1.85)**	**1.58 (1.14, 2.19)**	**0.65 (0.48, 0.88)***
ADH	0.69 (0.45, 1.05)	**0.33 (0.17, 0.66)***	**0.31 (0.15, 0.64)***	**0.48 (0.29, 0.79)***	0.81 (0.54, 1.23)	**0.27 (0.08, 0.89)**	0.66 (0.40, 1.09)	**0.33 (0.19, 0.58)***	**0.56 (0.35, 0.91)**	0.90 (0.53, 1.54)	**0.35 (0.13, 0.97)**	**0.27 (0.08, 0.86)**
FEA	0.88 (0.55, 1.39)	**0.45 (0.24, 0.85)***	**0.33 (0.16, 0.70)***	-	0.79 (0.50, 1.23)	0.41 (0.16, 1.09)	0.72 (0.42, 1.25)	**0.30 (0.16, 0.56)***	0.69 (0.43, 1.11)	0.85 (0.48, 1.52)	0.58 (0.24, 1.39)	**0.19 (0.05, 0.80)**
Radial scar/complex sclerosing	**0.60 (0.38, 0.97)**	0.98 (0.58, 1.66)	0.78 (0.46, 1.33)	0.87 (0.56, 1.35)	0.76 (0.50, 1.18)	0.88 (0.40, 1.92)	0.79 (0.45, 1.37)	0.64 (0.36, 1.14)	0.90 (0.56, 1.44)	1.08 (0.62, 1.87)	0.85 (0.40, 1.80)	0.42 (0.15, 1.17)
Intraductal papilloma	**0.51 (0.36, 0.73)***	**0.53 (0.34, 0.83)***	**0.63 (0.40, 0.97)**	**0.64 (0.45, 0.91)**	**0.55 (0.39, 0.79)***	0.65 (0.35, 1.21)	0.78 (0.51, 1.19)	**0.58 (0.37, 0.90)***	0.71 (0.49, 1.01)	1.05 (0.68, 1.61)	**0.33 (0.14, 0.77)**	0.94 (0.53, 1.65)
Any (binary)	**0.67 (0.55, 0.82)***	**0.67 (0.52, 0.87)***	**0.94 (0.75, 1.17)**	0.88 (0.73, 1.07)	**0.79 (0.65, 0.96)***	**0.49 (0.34, 0.69)***	1.04 (0.79, 1.38)	0.88 (0.67, 1.16)	**0.87 (0.77, 0.99)**	**1.34 (1.02, 1.75)**	**1.52 (1.08, 2.14)**	**0.67 (0.49, 0.91)***
Any (continuous)	**0.73 (0.65, 0.83)***	**0.71 (0.60, 0.83)***	**0.78 (0.67, 0.91)***	**0.80 (0.70, 0.91)***	**0.80 (0.71, 0.91)***	**0.60 (0.47, 0.77)***	0.94 (0.80, 1.11)	**0.77 (0.65, 0.91)***	0.98 (0.80, 1.20)	1.13 (0.97, 1.32)	0.97 (0.79, 1.19)	**0.63 (0.50, 0.80)***

Supplementary Table S5. Association between coexisting features and 10-year overall survival in 3,164 breast cancer patients with CNB reports (613 events). Hazard ratios (HRs) with 95% confidence intervals (CIs) are presented from Cox proportional hazards models evaluating the impact of coexisting breast features on 10-year overall survival among breast cancer patients. Statistically significant associations ($p < 0.05$) are indicated in bold, those that remain significant after Benjamini-Hochberg correction are denoted by *. a) Adjusted for age at diagnosis and year of diagnosis. b) Model one further adjusted for menstruation status, ethnicity, family history of cancer and parity. c) Model two further adjusted for tumour characteristics: stage and subtype.

	Number of patients	Number of events	Model 1[a] HR (95% CI)	Model 2[b] HR (95% CI)	Model 3[c] HR (95% CI)
Lobular neoplasia					
Lobular carcinoma in situ (LCIS)	60 (1.9%)	10 (16.7%)	0.96 (0.51, 1.79)	0.98 (0.53, 1.84)	1.07 (0.57, 2.01)
Atypical lobular hyperplasia (ALH)	11 (0.3%)	1 (9.1%)	0.52 (0.07, 3.68)	0.61 (0.09, 4.35)	1.11 (0.15, 7.93)
Any (binary)	66 (2.1%)	11 (2.0%)	0.97 (0.53, 1.76)	1.00 (0.55, 1.83)	1.12 (0.61, 2.03)
Any (continuous)	-	-	0.90 (0.52, 1.57)	0.94 (0.54, 1.65)	1.07 (0.60, 1.91)
Benign or non-atypical proliferative breast changes					
Fibroadenoma	109 (3.4%)	15 (13.8%)	0.79 (0.48, 1.33)	0.83 (0.50, 1.39)	0.96 (0.57, 1.61)
Calcification	630 (19.9%)	104 (16.5%)	0.88 (0.71, 1.09)	0.88 (0.71, 1.09)	0.95 (0.77, 1.18)
Cyst	84 (2.7%)	9 (10.7%)	0.55 (0.28, 1.06)	0.53 (0.27, 1.02)	0.70 (0.36, 1.35)
Apocrine metaplasia	117 (3.7%)	10 (8.5%)	**0.46 (0.25, 0.87)**	**0.49 (0.26, 0.92)**	**0.43 (0.23, 0.80)**
Columnar cell change	65 (2.1%)	5 (7.7%)	**0.41 (0.17, 1.00)**	0.44 (0.18, 1.07)	0.53 (0.22, 1.27)
Usual ductal hyperplasia (UDH)	62 (2.0%)	6 (9.7%)	0.66 (0.29, 1.47)	0.67 (0.30, 1.50)	0.68 (0.30, 1.52)
Sclerosing adenosis	136 (4.3%)	18 (13.2%)	0.71 (0.44, 1.14)	0.74 (0.46, 1.19)	0.87 (0.54, 1.39)
Fibrocystic change	125 (4.0%)	22 (17.6%)	0.98 (0.64, 1.50)	0.98 (0.64, 1.50)	1.03 (0.67, 1.59)
Any (binary)	942 (30.0%)	141 (15.0%)	**0.75 (0.62, 0.90)***	**0.75 (0.62, 0.91)***	**0.82 (0.68, 1.00)**
Any (continuous)	-	-	**0.84 (0.74, 0.95)***	**0.85 (0.75, 0.96)**	0.90 (0.79, 1.02)
Early neoplastic, papillary and complex sclerosing lesions					
Ductal carcinoma in situ (DCIS)	942 (29.8%)	145 (15.4%)	**0.79 (0.65, 0.95)***	**0.81 (0.67, 0.98)**	0.94 (0.78, 1.14)
Atypical ductal hyperplasia (ADH)	21 (0.7%)	2 (9.5%)	0.41 (0.10, 1.63)	0.45 (0.11, 1.81)	0.53 (0.13, 2.12)
Flat epithelial atypia (FEA)	9 (0.3%)	0 (0.0%)	-	-	-
Radial scar or complex sclerosing lesion	19 (0.6%)	1 (5.3%)	0.33 (0.05, 2.37)	0.33 (0.05, 2.35)	0.36 (0.05, 2.58)
Intraductal papilloma	70 (2.2%)	5 (7.1%)	**0.36 (0.15, 0.86)**	**0.37 (0.15, 0.90)**	0.44 (0.18, 1.05)
Any (binary)	993 (31.4%)	149 (15.0%)	**0.75 (0.63, 0.91)***	**0.78 (0.65, 0.94)**	0.91 (0.75, 1.10)
Any (continuous)	-	-	**0.74 (0.63, 0.88)***	**0.77 (0.64, 0.91)***	0.88 (0.74, 1.05)

Supplementary Table S6. Association between coexisting features and 10-year overall survival in 1,756 breast cancer cases with reports from excision procedures, diagnosed before 2010. Hazard ratios (HRs) with 95% confidence intervals (CIs) are presented from Cox proportional hazards models evaluating the impact of coexisting breast features on 10-year overall survival among breast cancer patients. Statistically significant associations ($p < 0.05$) are indicated in bold, those that remain significant after Benjamini-Hochberg correction are denoted by *. a) Adjusted for age at diagnosis and year of diagnosis. b) Model one further adjusted for menstruation status, ethnicity, family history of cancer and parity. c) Model two further adjusted for tumour characteristics: stage and subtype.

	Number of patients	Number of events	Model 1[a] HR (95% CI)	Model 2[b] HR (95% CI)	Model 3[c] HR (95% CI)
Lobular neoplasia					
Lobular carcinoma in situ (LCIS)	86 (4.9%)	18 (20.9%)	1.05 (0.65, 1.69)	1.01 (0.63, 1.63)	1.08 (0.67, 1.75)
Atypical lobular hyperplasia (ALH)	31 (1.8%)	4 (12.9%)	0.69 (0.26, 1.84)	0.71 (0.26, 1.90)	0.83 (0.30, 2.25)
Any (binary)	102 (5.8%)	19 (18.6%)	0.95 (0.60, 1.51)	0.92 (0.58, 1.47)	1.04 (0.65, 1.67)
Any (continuous)	-	-	0.96 (0.66, 1.41)	0.95 (0.64, 1.40)	1.02 (0.69, 1.49)
Benign or non-atypical proliferative breast changes					
Fibroadenoma	152 (8.7%)	25 (16.4%)	0.88 (0.58, 1.32)	0.90 (0.60, 1.36)	0.97 (0.64, 1.47)
Calcification	321 (18.3%)	47 (14.6%)	**0.72 (0.53, 0.98)**	**0.71 (0.52, 0.97)**	0.89 (0.65, 1.22)
Cyst	194 (11.0%)	25 (12.9%)	0.70 (0.46, 1.05)	0.70 (0.46, 1.06)	0.74 (0.49, 1.12)
Apocrine metaplasia	135 (7.7%)	16 (11.9%)	0.62 (0.37, 1.02)	0.63 (0.38, 1.04)	0.71 (0.43, 1.18)
Columnar cell change	95 (5.4%)	8 (8.4%)	**0.47 (0.23, 0.95)**	**0.49 (0.24, 0.99)**	0.50 (0.25, 1.03)
Usual ductal hyperplasia (UDH)	122 (6.9%)	6 (4.9%)	**0.24 (0.11, 0.55)***	**0.25 (0.11, 0.56)***	**0.34 (0.15, 0.77)**
Sclerosing adenosis	128 (7.3%)	16 (12.5%)	0.70 (0.42, 1.16)	0.72 (0.44, 1.20)	0.92 (0.55, 1.53)
Fibrocystic change	415 (23.6%)	59 (14.2%)	**0.71 (0.54, 0.94)**	0.76 (0.57, 1.01)	0.87 (0.65, 1.16)
Any (binary)	743 (42.3%)	117 (15.7%)	**0.72 (0.57, 0.90)***	**0.73 (0.58, 0.91)***	0.85 (0.67, 1.06)
Any (continuous)	-	-	**0.84 (0.76, 0.93)***	**0.85 (0.77, 0.94)***	**0.90 (0.82, 1.00)**
Early neoplastic, papillary and complex sclerosing lesions					
Ductal carcinoma in situ (DCIS)	1074 (61.2%)	191 (17.8%)	0.84 (0.68, 1.05)	0.86 (0.69, 1.07)	1.02 (0.81, 1.28)
Atypical ductal hyperplasia (ADH)	60 (3.4%)	8 (13.3%)	0.69 (0.34, 1.40)	0.72 (0.35, 1.45)	0.94 (0.46, 1.92)
Flat epithelial atypia (FEA)	8 (0.5%)	1 (12.5%)	0.71 (0.10, 5.08)	0.70 (0.10, 5.03)	0.80 (0.11, 5.76)
Radial scar or complex sclerosing lesion	43 (2.4%)	9 (20.9%)	1.08 (0.56, 2.09)	1.06 (0.54, 2.05)	1.10 (0.57, 2.14)
Intraductal papilloma	57 (3.2%)	11 (19.3%)	1.04 (0.57, 1.89)	1.05 (0.57, 1.93)	1.12 (0.60, 2.07)
Any (binary)	1108 (63.1%)	197 (17.8%)	0.84 (0.67, 1.04)	0.85 (0.68, 1.06)	0.99 (0.79, 1.25)
Any (continuous)	-	-	0.88 (0.73, 1.05)	0.89 (0.74, 1.06)	1.02 (0.85, 1.23)

Supplementary Table S7. Association between coexisting features and 10-year overall survival in 2,232 breast cancer cases with reports from excision procedures, diagnosed 2010 and after. Hazard ratios (HRs) with 95% confidence intervals (CIs) are presented from Cox proportional hazards models evaluating the impact of coexisting breast features on 10-year overall survival among breast cancer patients. Statistically significant associations ($p < 0.05$) are indicated in bold, those that remain significant after Benjamini-Hochberg correction are denoted by *. a) Adjusted for age at diagnosis and year of diagnosis. b) Model one further adjusted for menstruation status, ethnicity, family history of cancer and parity. c) Model two further adjusted for tumour characteristics: stage and subtype.

	Number of patients	Number of events	Model 1[a] HR (95% CI)	Model 2[b] HR (95% CI)	Model 3[c] HR (95% CI)
Lobular neoplasia					
Lobular carcinoma in situ (LCIS)	160 (7.2%)	18 (11.2%)	1.01 (0.62, 1.63)	1.01 (0.62, 1.63)	1.17 (0.72, 1.90)
Atypical lobular hyperplasia (ALH)	49 (2.2%)	4 (8.2%)	0.86 (0.32, 2.31)	0.79 (0.29, 2.12)	0.93 (0.34, 2.52)
Any (binary)	185 (8.3%)	22 (11.9%)	1.11 (0.72, 1.71)	1.10 (0.71, 1.71)	1.23 (0.79, 1.92)
Any (continuous)	-	-	0.98 (0.66, 1.45)	0.96 (0.65, 1.43)	1.10 (0.73, 1.65)
Benign or non-atypical proliferative breast changes					
Fibroadenoma	339 (15.2%)	42 (12.4%)	0.98 (0.71, 1.37)	0.92 (0.66, 1.27)	0.94 (0.67, 1.31)
Calcification	699 (31.3%)	69 (9.9%)	**0.74 (0.56, 0.97)**	**0.71 (0.54, 0.93)**	**0.72 (0.55, 0.95)**
Cyst	432 (19.4%)	49 (11.3%)	0.91 (0.67, 1.24)	0.84 (0.62, 1.16)	0.88 (0.64, 1.21)
Apocrine metaplasia	288 (12.9%)	32 (11.1%)	0.89 (0.62, 1.29)	0.89 (0.61, 1.29)	1.00 (0.69, 1.46)
Columnar cell change	253 (11.3%)	28 (11.1%)	0.93 (0.63, 1.38)	0.92 (0.62, 1.36)	1.06 (0.71, 1.58)
Usual ductal hyperplasia (UDH)	291 (13.0%)	39 (13.4%)	1.09 (0.77, 1.52)	1.03 (0.74, 1.45)	1.17 (0.83, 1.66)
Sclerosing adenosis	245 (11.0%)	30 (12.2%)	1.07 (0.73, 1.57)	1.06 (0.73, 1.56)	1.21 (0.82, 1.77)
Fibrocystic change	627 (28.1%)	69 (11.0%)	0.91 (0.70, 1.20)	0.91 (0.69, 1.19)	0.99 (0.75, 1.30)
Any (binary)	1324 (59.3%)	158 (12.0%)	0.90 (0.71, 1.13)	0.86 (0.68, 1.09)	0.90 (0.71, 1.14)
Any (continuous)	-	-	0.96 (0.89, 1.04)	0.95 (0.88, 1.02)	0.98 (0.91, 1.06)
Early neoplastic, papillary and complex sclerosing lesions					
Ductal carcinoma in situ (DCIS)	1375 (61.6%)	176 (12.8%)	1.00 (0.79, 1.27)	0.98 (0.77, 1.25)	1.10 (0.87, 1.41)
Atypical ductal hyperplasia (ADH)	110 (4.9%)	6 (5.5%)	**0.41 (0.18, 0.92)**	**0.41 (0.18, 0.91)**	0.49 (0.22, 1.10)
Flat epithelial atypia (FEA)	100 (4.5%)	7 (7.0%)	0.60 (0.28, 1.27)	0.64 (0.30, 1.38)	0.81 (0.37, 1.75)
Radial scar or complex sclerosing lesion	105 (4.7%)	8 (7.6%)	0.60 (0.30, 1.22)	0.61 (0.30, 1.23)	0.59 (0.29, 1.21)
Intraductal papilloma	183 (8.2%)	30 (16.4%)	1.22 (0.84, 1.78)	1.22 (0.83, 1.78)	1.46 (0.99, 2.15)
Any (binary)	1480 (66.3%)	196 (13.2%)	1.10 (0.86, 1.41)	1.10 (0.85, 1.41)	1.24 (0.97, 1.60)
Any (continuous)	-	-	0.93 (0.79, 1.08)	0.92 (0.78, 1.08)	1.02 (0.87, 1.20)

Supplementary Table S8. Association between coexisting features and 10-year overall survival in 1,604 breast cancer cases with reports from excision procedures diagnosed with Stage I breast cancer. Hazard ratios (HRs) with 95% confidence intervals (CIs) are presented from Cox proportional hazards models evaluating the impact of coexisting breast features on 10-year overall survival among breast cancer patients. Statistically significant associations ($p < 0.05$) are indicated in bold, those that remain significant after Benjamini-Hochberg correction are denoted by *. a) Adjusted for age at diagnosis and year of diagnosis. b) Model one further adjusted for menstruation status, ethnicity, family history of cancer and parity. c) Model two further adjusted for tumour characteristics: subtype.

	Number of patients	Number of events	Model 1[a] HR (95% CI)	Model 2[b] HR (95% CI)	Model 3[c] HR (95% CI)
Lobular neoplasia					
Lobular carcinoma in situ (LCIS)	109 (6.8%)	6 (5.5%)	0.81 (0.35, 1.83)	0.79 (0.34, 1.80)	0.78 (0.34, 1.80)
Atypical lobular hyperplasia (ALH)	52 (3.2%)	2 (3.8%)	0.72 (0.18, 2.92)	0.70 (0.17, 2.87)	0.71 (0.17, 2.92)
Any (binary)	134 (8.4%)	8 (6.0%)	0.92 (0.45, 1.88)	0.91 (0.44, 1.87)	0.91 (0.44, 1.88)
Any (continuous)	-	-	0.82 (0.43, 1.55)	0.81 (0.43, 1.53)	0.81 (0.43, 1.53)
Benign or non-atypical proliferative breast changes					
Fibroadenoma	191 (11.9%)	14 (7.3%)	0.98 (0.56, 1.70)	0.96 (0.55, 1.67)	0.96 (0.55, 1.68)
Calcification	510 (31.8%)	32 (6.3%)	0.82 (0.55, 1.23)	0.80 (0.53, 1.20)	0.82 (0.54, 1.23)
Cyst	257 (16.0%)	14 (5.4%)	0.69 (0.39, 1.20)	0.68 (0.39, 1.18)	0.67 (0.38, 1.17)
Apocrine metaplasia	215 (13.4%)	10 (4.7%)	0.56 (0.29, 1.07)	0.57 (0.30, 1.08)	0.53 (0.28, 1.03)
Columnar cell change	180 (11.2%)	9 (5.0%)	0.73 (0.37, 1.44)	0.76 (0.38, 1.50)	0.77 (0.39, 1.52)
Usual ductal hyperplasia (UDH)	211 (13.2%)	18 (8.5%)	1.26 (0.77, 2.08)	1.25 (0.76, 2.07)	1.25 (0.76, 2.07)
Sclerosing adenosis	189 (11.8%)	14 (7.4%)	1.07 (0.61, 1.87)	1.09 (0.63, 1.90)	1.12 (0.64, 1.96)
Fibrocystic change	491 (30.6%)	38 (7.7%)	1.12 (0.76, 1.64)	1.15 (0.79, 1.69)	1.18 (0.80, 1.74)
Any (binary)	914 (57.0%)	65 (7.1%)	0.86 (0.61, 1.22)	0.86 (0.61, 1.22)	0.86 (0.60, 1.21)
Any (continuous)	-	-	0.96 (0.86, 1.07)	0.96 (0.86, 1.07)	0.96 (0.86, 1.08)
Early neoplastic, papillary and complex sclerosing lesions					
Ductal carcinoma in situ (DCIS)	1073 (66.9%)	84 (7.8%)	0.99 (0.69, 1.43)	0.94 (0.65, 1.37)	0.97 (0.67, 1.40)
Atypical ductal hyperplasia (ADH)	93 (5.8%)	6 (6.5%)	0.91 (0.40, 2.06)	0.89 (0.39, 2.03)	0.91 (0.40, 2.09)
Flat epithelial atypia (FEA)	49 (3.1%)	1 (2.0%)	0.35 (0.05, 2.54)	0.38 (0.05, 2.75)	0.39 (0.05, 2.83)
Radial scar or complex sclerosing lesion	74 (4.6%)	8 (10.8%)	1.39 (0.68, 2.84)	1.57 (0.76, 3.24)	1.63 (0.79, 3.36)
Intraductal papilloma	129 (8.0%)	16 (12.4%)	**1.69 (1.00, 2.86)**	**1.71 (1.01, 2.90)**	**1.75 (1.03, 2.98)**
Any (binary)	1138 (70.9%)	94 (8.3%)	1.21 (0.81, 1.79)	1.17 (0.78, 1.74)	1.20 (0.81, 1.79)
Any (continuous)	-	-	1.08 (0.86, 1.37)	1.08 (0.85, 1.37)	1.10 (0.87, 1.40)

Supplementary Table S9. Association between coexisting features and 10-year overall survival in 1,634 breast cancer cases with reports from excision procedures diagnosed with Stage II breast cancer. Hazard ratios (HRs) with 95% confidence intervals (CIs) are presented from Cox proportional hazards models evaluating the impact of coexisting breast features on 10-year overall survival among breast cancer patients. Statistically significant associations ($p < 0.05$) are indicated in bold, those that remain significant after Benjamini-Hochberg correction are denoted by *. a) Adjusted for age at diagnosis and year of diagnosis. b) Model one further adjusted for menstruation status, ethnicity, family history of cancer and parity. c) Model two further adjusted for tumour characteristics: subtype.

	Number of patients	Number of events	Model 1[a] HR (95% CI)	Model 2[b] HR (95% CI)	Model 3[c] HR (95% CI)
Lobular neoplasia					
Lobular carcinoma in situ (LCIS)	94 (5.8%)	14 (14.9%)	1.04 (0.60, 1.78)	1.08 (0.63, 1.87)	1.15 (0.66, 2.00)
Atypical lobular hyperplasia (ALH)	20 (1.2%)	5 (25.0%)	1.92 (0.79, 4.65)	2.13 (0.86, 5.27)	2.18 (0.88, 5.41)
Any (binary)	116 (7.1%)	14 (14.9%)	1.15 (0.70, 1.89)	1.22 (0.74, 2.00)	1.30 (0.78, 2.15)
Any (continuous)	-	-	1.16 (0.75, 1.78)	1.22 (0.79, 1.87)	1.28 (0.83, 1.98)
Benign or non-atypical proliferative breast changes					
Fibroadenoma	192 (11.8%)	26 (13.5%)	0.94 (0.62, 1.41)	0.93 (0.62, 1.41)	0.97 (0.64, 1.46)
Calcification	351 (21.5%)	47 (13.4%)	0.92 (0.67, 1.27)	0.91 (0.66, 1.26)	0.93 (0.68, 1.29)
Cyst	255 (15.6%)	33 (12.9%)	0.96 (0.67, 1.39)	0.98 (0.67, 1.42)	0.99 (0.68, 1.44)
Apocrine metaplasia	157 (9.6%)	22 (14.0%)	1.08 (0.70, 1.67)	1.06 (0.68, 1.65)	1.06 (0.68, 1.65)
Columnar cell change	120 (7.3%)	17 (14.2%)	1.13 (0.69, 1.86)	1.17 (0.71, 1.92)	1.22 (0.74, 2.00)
Usual ductal hyperplasia (UDH)	160 (9.8%)	15 (9.4%)	0.63 (0.37, 1.07)	0.62 (0.37, 1.05)	0.64 (0.38, 1.08)
Sclerosing adenosis	135 (8.3%)	21 (15.6%)	1.25 (0.80, 1.96)	1.24 (0.79, 1.95)	1.32 (0.84, 2.09)
Fibrocystic change	399 (24.4%)	51 (12.8%)	0.91 (0.66, 1.23)	0.91 (0.66, 1.24)	0.91 (0.67, 1.24)
Any (binary)	803 (49.1%)	109 (13.6%)	0.89 (0.69, 1.15)	0.90 (0.69, 1.16)	0.93 (0.72, 1.21)
Any (continuous)	-	-	0.98 (0.89, 1.07)	0.98 (0.89, 1.07)	0.99 (0.90, 1.08)
Early neoplastic, papillary and complex sclerosing lesions					
Ductal carcinoma in situ (DCIS)	928 (56.8%)	137 (14.8%)	1.02 (0.79, 1.32)	1.02 (0.78, 1.32)	1.10 (0.84, 1.44)
Atypical ductal hyperplasia (ADH)	62 (3.8%)	7 (11.3%)	0.77 (0.36, 1.63)	0.81 (0.38, 1.72)	0.85 (0.40, 1.82)
Flat epithelial atypia (FEA)	44 (2.7%)	2 (4.5%)	0.32 (0.08, 1.30)	0.35 (0.09, 1.42)	0.39 (0.09, 1.57)
Radial scar or complex sclerosing lesion	45 (2.8%)	5 (11.1%)	0.99 (0.41, 2.39)	0.95 (0.39, 2.31)	1.00 (0.41, 2.42)
Intraductal papilloma	71 (4.3%)	8 (11.3%)	0.67 (0.33, 1.37)	0.68 (0.34, 1.38)	0.71 (0.35, 1.44)
Any (binary)	979 (59.9%)	143 (14.6%)	1.00 (0.77, 1.29)	1.00 (0.77, 1.30)	1.08 (0.83, 1.42)
Any (continuous)	-	-	0.92 (0.76, 1.12)	0.92 (0.76, 1.13)	0.98 (0.80, 1.20)

Supplementary Table S10. Association between coexisting features and 10-year overall survival in 750 breast cancer cases with reports from excision procedures diagnosed with Stage III breast cancer. Hazard ratios (HRs) with 95% confidence intervals (CIs) are presented from Cox proportional hazards models evaluating the impact of coexisting breast features on 10-year overall survival among breast cancer patients. Statistically significant associations ($p < 0.05$) are indicated in bold, those that remain significant after Benjamini-Hochberg correction are denoted by *. a) Adjusted for age at diagnosis and year of diagnosis. b) Model one further adjusted for menstruation status, ethnicity, family history of cancer and parity. c) Model two further adjusted for tumour characteristics: subtype.

			Model 1[a]	Model 2[b]	Model 3[c]
	Number of patients	Number of events	HR (95% CI)	HR (95% CI)	HR (95% CI)
Lobular neoplasia					
Lobular carcinoma in situ (LCIS)	43 (5.7%)	16 (37.2%)	1.19 (0.72, 1.98)	1.21 (0.73, 2.02)	1.28 (0.76, 2.14)
Atypical lobular hyperplasia (ALH)	8 (1.1%)	1 (12.5%)	0.36 (0.05, 2.60)	0.32 (0.04, 2.30)	0.39 (0.05, 2.86)
Any (binary)	47 (6.3%)	16 (34.0%)	1.09 (0.66, 1.82)	1.09 (0.65, 1.82)	1.16 (0.69, 1.95)
Any (continuous)	-	-	1.04 (0.67, 1.63)	1.03 (0.66, 1.62)	1.11 (0.70, 1.75)
Benign or non-atypical proliferative breast changes					
Fibroadenoma	108 (14.4%)	27 (25.0%)	0.71 (0.48, 1.06)	0.74 (0.49, 1.11)	0.83 (0.55, 1.25)
Calcification	159 (21.2%)	37 (23.3%)	**0.64 (0.45, 0.90)**	**0.64 (0.45, 0.91)**	**0.66 (0.46, 0.94)**
Cyst	114 (15.2%)	27 (23.7%)	0.70 (0.47, 1.04)	0.72 (0.48, 1.08)	0.69 (0.46, 1.04)
Apocrine metaplasia	51 (6.8%)	16 (31.4%)	0.96 (0.58, 1.60)	0.99 (0.59, 1.64)	1.03 (0.62, 1.73)
Columnar cell change	48 (6.4%)	10 (20.8%)	0.60 (0.32, 1.13)	0.66 (0.35, 1.26)	0.63 (0.33, 1.19)
Usual ductal hyperplasia (UDH)	42 (5.6%)	12 (28.6%)	0.93 (0.52, 1.66)	0.90 (0.50, 1.62)	0.95 (0.53, 1.71)
Sclerosing adenosis	49 (6.5%)	11 (22.4%)	0.72 (0.39, 1.33)	0.75 (0.41, 1.38)	0.77 (0.42, 1.41)
Fibrocystic change	152 (20.3%)	39 (25.7%)	0.79 (0.56, 1.11)	0.81 (0.57, 1.14)	0.83 (0.59, 1.17)
Any (binary)	350 (46.7%)	101 (28.9%)	**0.77 (0.60, 0.99)**	0.78 (0.60, 1.01)	0.80 (0.62, 1.04)
Any (continuous)	-	-	**0.87 (0.78, 0.97)**	**0.88 (0.79, 0.98)**	**0.89 (0.80, 0.99)**
Early neoplastic, papillary and complex sclerosing lesions					
Ductal carcinoma in situ (DCIS)	448 (59.7%)	146 (32.6%)	0.89 (0.69, 1.14)	0.92 (0.72, 1.18)	1.01 (0.78, 1.31)
Atypical ductal hyperplasia (ADH)	15 (2.0%)	1 (6.7%)	0.16 (0.02, 1.14)	0.16 (0.02, 1.15)	0.18 (0.03, 1.31)
Flat epithelial atypia (FEA)	15 (2.0%)	5 (33.3%)	0.97 (0.40, 2.36)	1.07 (0.43, 2.64)	1.17 (0.47, 2.92)
Radial scar or complex sclerosing lesion	29 (3.9%)	4 (13.8%)	**0.34 (0.13, 0.91)**	**0.30 (0.11, 0.82)**	**0.33 (0.12, 0.89)**
Intraductal papilloma	40 (5.3%)	17 (42.5%)	1.53 (0.93, 2.51)	1.62 (0.98, 2.70)	**1.74 (1.05, 2.90)**
Any (binary)	471 (62.8%)	156 (33.1%)	0.92 (0.72, 1.19)	0.95 (0.73, 1.22)	1.05 (0.81, 1.37)
Any (continuous)	-	-	0.88 (0.73, 1.07)	0.90 (0.74, 1.08)	0.97 (0.80, 1.17)

Supplementary Table S11. Association between tumor stage and presence/number of breast features, using 3,988 breast cancer cases with reports from excision procedures.

Model type	Predictor (ref Stage I)	Estimate	95% confidence interval	p-value
Logistic (binary)	Stage II	0.597	(0.501, 0.710)	6.35E-09
	Stage III	0.681	(0.548, 0.847)	5.28E-04
Poisson (count)	Stage II	0.779	(0.743, 0.817)	8.52E-25
	Stage III	0.739	(0.694, 0.786)	2.61E-21

Supplementary Figure S1. Flowchart of how analytical datasets were derived.

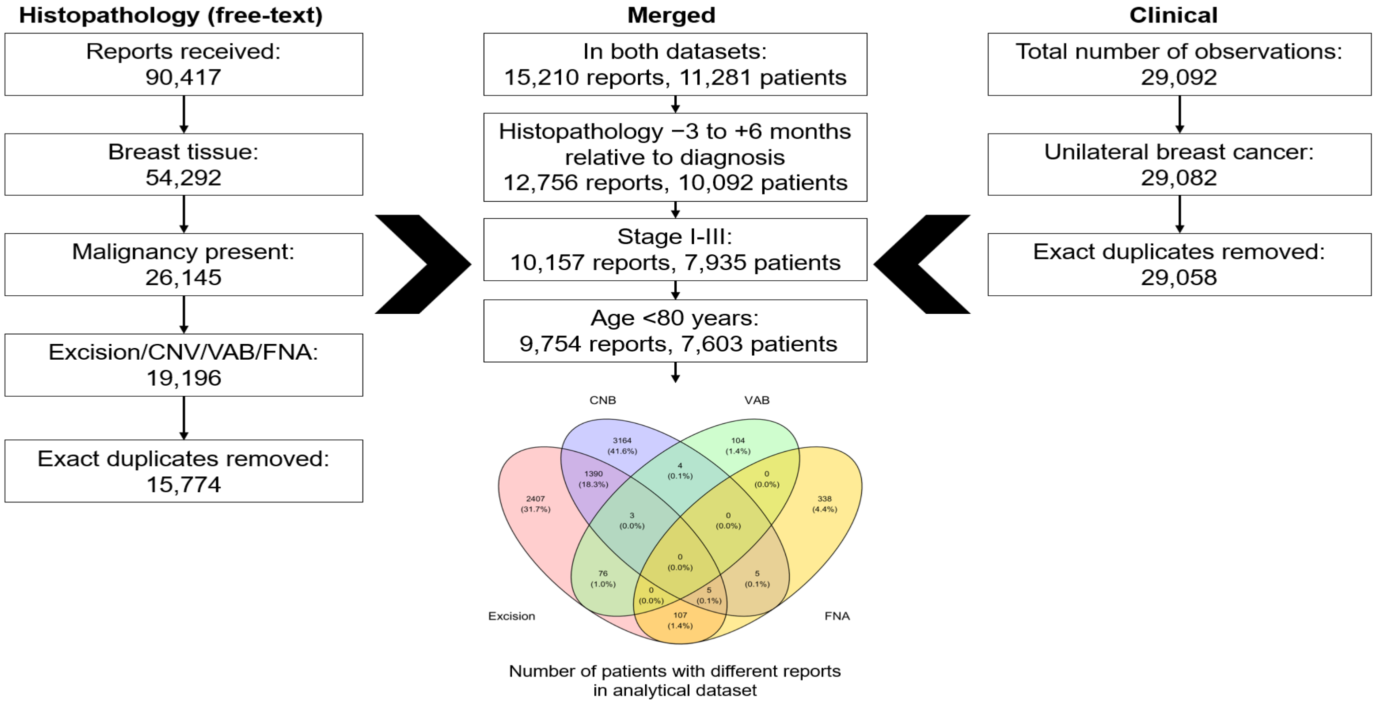

Supplementary Figure S2. Distribution of procedure date relative to diagnosis date.

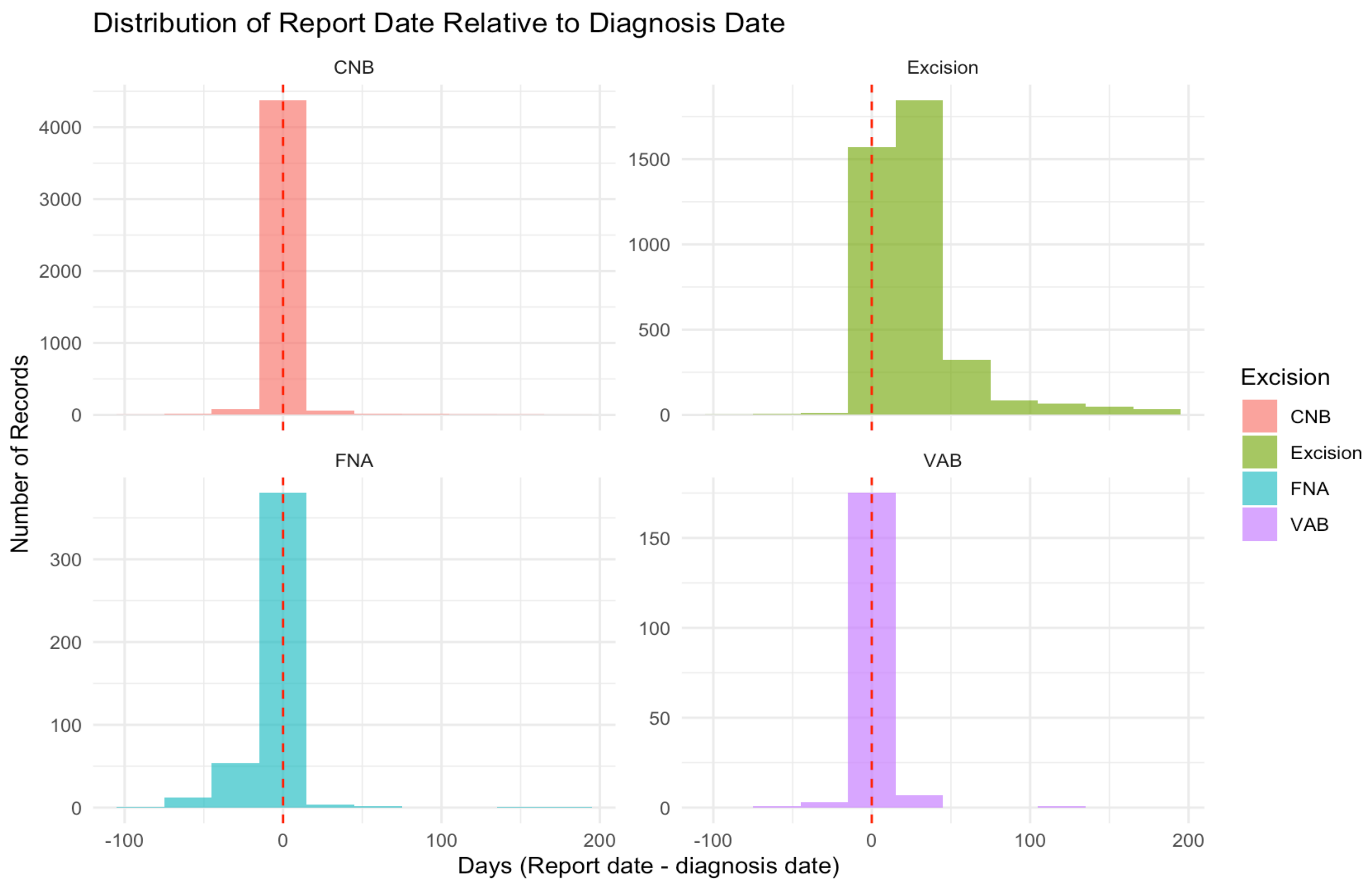

Supplementary Figure S3. A) Cluster membership of breast features based on Pearson correlation hierarchical clustering for 3,988 records from excisions. **B)** Sensitivity analysis: Jaccard distance with complete linkage (Adjusted Rand Index vs. Panel A = 0.574). The lobular track (LCIS, ALH) is stable across both methods. Pre-malignant and high-risk ductal lesions (DCIS, intraductal papilloma) shift from the high-risk ductal cluster (A) to the benign cluster (B), reflecting Jaccard's exclusion of joint absences which masks the epidemiological rarity these lesions share across benign screening records.

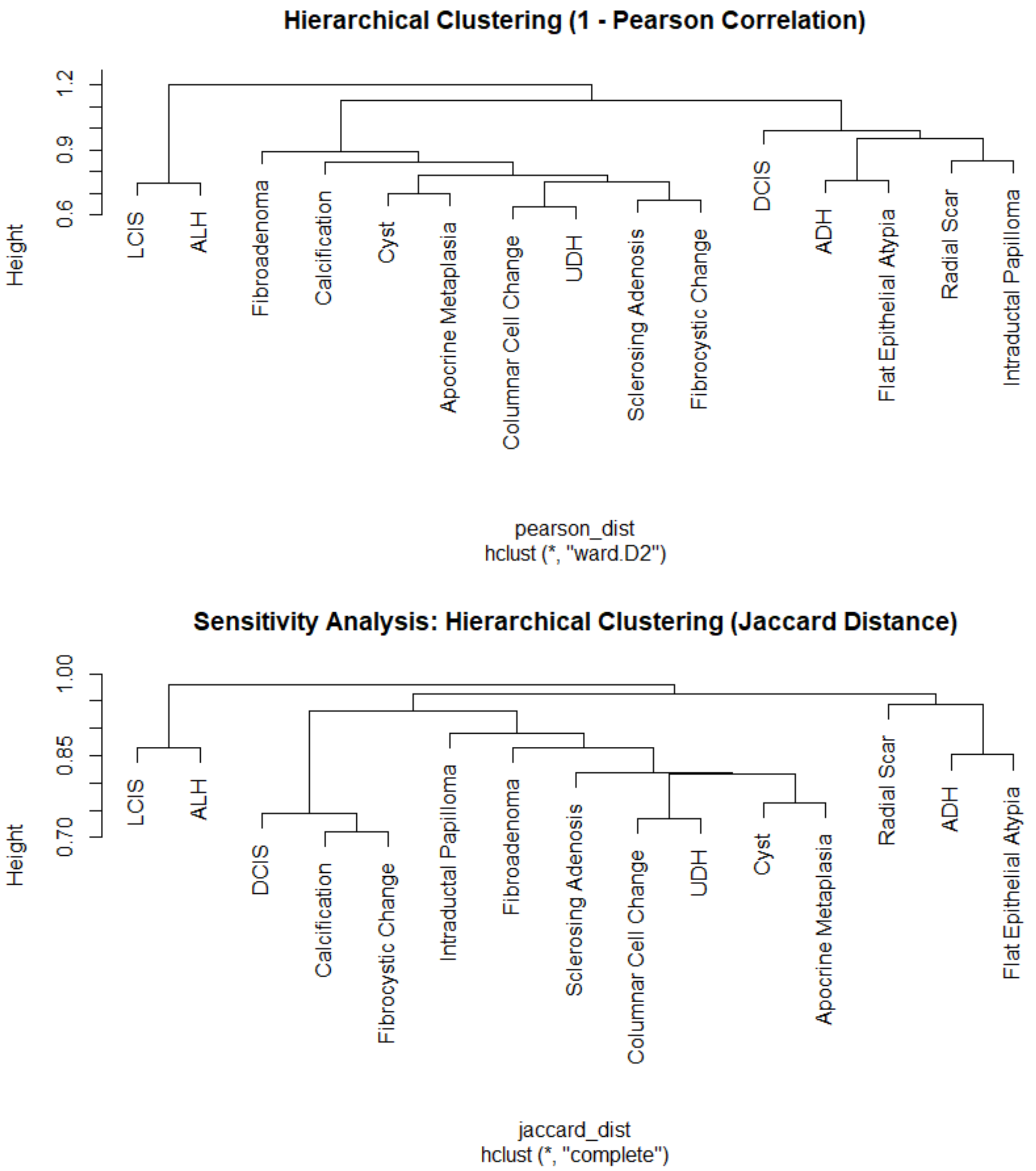

Supplement: Supplementary file 1 [file cancers-18-01965-s001.zip › Supplementary Material S.pdf]
